# Spatiotemporal patterns of healthy life expectancy and the effects of health financing in West African countries, 1995-2019: A Spatial Panel Modelling Study

**DOI:** 10.7189/jogh.13.04123

**Published:** 2023-10-20

**Authors:** Meng Zeng, Lu Niu

**Affiliations:** Department of Social Medicine and Health Management, Xiangya School of Public Health, Central South University, Changsha, China

## Abstract

**Background:**

Health financing produce a broad range of healthy life expectancy (HLE) disparities. In West Africa, limited research exists on the association between health financing and HLE at ecological level during a consecutive period of time from the spatial perspectives. This study aimed to determine the existence, quantify the magnitude, and interpret the association between health financing and HLE.

**Methods:**

A Dynamic Spatial Durbin model was used to explain the association between HLE and health financing level and structure during 1995-2019 in West Africa. Spatial spillover effects were introduced to interpret the direct and indirect effects caused by health financing level and structure on HLE during the long and short terms.

**Results:**

Spatial dependence and clustering on HLE were observed in West Africa. Although the overall level of total health spending, government health spending, out-of-pocket health spending, and development assistance for health (DAH) increased from 1995 to 2019, government health spending per person experienced a declining trend. Out-of-pocket health spending per total health spending was the highest among other sources of health financing, decreasing from 57% during 1995-1999 to 42% during 2015-2019. Total health spending and out-of-pocket health spending affected HLE positively and negatively in the long term, respectively. Government health spending and prepaid private health spending per person had positive effects on local and adjacent country HLE in the short-term, while DAH had negative effects on the same. The short-term spatial spillover effects of government health spending, DAH, and prepaid private health spending per person were more pronounced than the long-term effects.

**Conclusions:**

Spatial variations of HLE existed at country-level in West Africa. Health financing regarding government, non-government, as well as external assistance not only affected HLE disparities at local scale but also among nearby countries. Policymakers should optimise supportive health financing transition policies and narrow the national gap to reduce health disparities and increase HLE. Externalities of policy of those health financing proxies should be took into consideration to promote health equity to improve global health governance.

The United Nation (UN) has recognised life expectancy as a critical parameter for assessing the health status of a population. The World Health Organization (WHO) shares the same view and has also adopted life expectancy as a key metric for measuring the health of a population. In 1997, the WHO emphasised the importance of healthy life expectancy (HLE) over life expectancy alone, stating that a longer life without quality of life is meaningless [1]. HLE provides a more comprehensive assessment of health by taking years of life lost into account due to disease and disability. It is currently the most widely used health measurement index, developed by the Institute for Health Metrics and Evaluation (IHME) at the University of Washington [1]. However, significant disparities in life expectancy and health outcomes exist among different populations and regions. The African Union Agenda 2063 set out health goals, including 75 years of life expectancy at birth in African countries by 2063 [2]. In 2019, life expectancy in West region of Sub-Saharan Africa was 63.8 years and healthy life expectancy was 57.5 years, the lowest in the world [3]. Additionally, it is also home to the world's greatest concentration of relative poverty hindering population health in West Africa.

Previous studies have primarily focused on regional differences, dynamic changes, and influencing factors of life expectancy, with limited literature investigating the influencing factors of HLE. While most scholars and researchers have assessed the association between health financing and health outcomes based on the Grossman health needs model [4], there is no consistent conclusion on the impact of health financing on health outcomes. Existing literature suggests that national health financing systems play a crucial role in health outcomes, with a strong negative correlation between increased health spending and life expectancy at birth in cross-country studies [5]. However, previous studies have also shown that health spending is not the key determinant of health outcomes. Public health spending and out-of-pocket spending by individuals have been positively correlated with life expectancy [6-10]. Furthermore, a study in the Middle East and North Africa revealed that some countries spend more on health expenditure but still have lower life expectancy [11]. Inconsistent conclusions regarding the correlation between health spending and life expectancy have led researchers to perform geographical weighted regression models, spatial Durbin models, and other methods to analyse the effects of life expectancy, reporting the existence of spatial spillover effects between health spending and life expectancy from a spatial perspective [12]. Nevertheless, previous studies have mainly considered the level of health financing from a spatial perspective and have rarely taken the structure of health financing into account or the impact of changes on life expectancy. Over the past few decades, global health spending has grown significantly, with significant disparities between per capita health spending in low- and middle-income countries and high-income countries [13]. Presently, many countries are still unable to fund a basic set of health services, and the proportion of development assistance for health is rising in low-income West African countries [14]. Health financing structures in West African countries have changed over time, and government health spending has a more profound impact on life expectancy than health spending from other sources [15]. Consequently, the impact of health spending on population health may vary depending on the sources of health financing. Combining both the temporal and spatial dimensions, it is crucial to explore the impact of the level and structure of health financing on HLE in West African countries.

The aim of this study is to address existing gaps in research and examine the spatiotemporal distribution of Healthy Life Expectancy (HLE) in West African countries. Furthermore, we will analyse the effects of health financing transition, such as level and structure, by utilising data from the IHME during the period of 1995-2019. The results will provide insight into the changes in HLE on a country level over time and evaluate the impact of health financing transition on the short and long-term trends of HLE. The findings of our study will be valuable for policymakers and health advocates to improve global health governance and reduce health disparities by ensuring adequate health financing resources are available for health.

## METHODS

### Study area and data source

In this ecological study, we focused on 15 countries in West Africa, which are members of the Economic Community of West African States (ECOWAS). These countries include Benin, Burkina Faso, Cote d’Ivoire, Cape Verde, Gambia, Ghana, Guinea, Guinea-Bissau, Liberia, Mali, Niger, Nigeria, Sierra Leone, Senegal, and Togo. We collected data on HLE from 1995 to 2019 for each country from the Global Burden of Diseases, Injuries, and Risk Factors Study (GBD) 2019 database (https://ghdx.healthdata.org/gbd-2019). The GBD 2019 is a comprehensive health loss study using uniform and comparable methodologies based on reliable and representative data from multiple sources from 1990 to 2019 for 204 countries and territories by sex and age, systematically analysing and evaluating disease burden. Additionally, we used health financing data obtained from the IHME’s Global Health Financing (GHF)2021 database (https://ghdx.healthdata.org/series/financing-global-health-fgh). The GHF2021 database provides health spending data collected from various sources including the WHO’s Global Health Expenditure Database, national health accounts and project-level data on development assistance for health.

### Indicators of health financing

In our research, we focused on indicators of health financing, which are used to measure a country's spending on health care with the primary aim of maintaining or improving health. This spending includes, for example, provision of preventive, curative, and palliative medicine, but not expenses related to water and sanitation, humanitarian aid, or distal health determinants [16]. We also examined the concept of health financing transition, which refers to the shift that countries experience from low health spending primarily funded out-of-pocket to high health spending primarily funded through pooled resources. To assess the level and structure of health financing, we used the GHF2021 to obtain health spending data for level. In addition, we included four indicators of health financing structure at the country level: Government Health Spending per Total Health Spending, Prepaid Private Health Spending per Total Health Spending, Out-of-pocket Health Spending per Total Health Spending and Development Assistance for Health per Total Health Spending. According to GHF2021, we define:

i) Total Health Spending refers to the sum of Government Health Spending, Prepaid Private Health Spending, Out-of-pocket Health Spending and Development Assistance for Health.

ii) Government Health Spending refers to spending for health care that is derived from domestic sources and is mutually exclusive from out-of-pocket, prepaid private and DAH spending.

iii) Prepaid Private Health Spending refers to health spending sources from non-public programs that are funded prior to obtaining health care, such as private health insurance and services provided for free by non-governmental agencies.

iv) Out-of-pocket Health Spending refers to payments made by individuals for health maintenance, restoration, or enhancement at or after the time of health care delivery, including health insurance copayments or payments devoted to deductibles.

v) Development Assistance for Health (DAH) refers to financial and in-kind resources that are transferred through international development agencies to low- and middle-income countries with the primary purpose of maintaining or improving health.

### Statistical methods

#### Statistical description

The health financing indicators were statistically described by using the D'Agostino-Pearson normality test to determine the distribution type of each indicator (Table S1 in the **Online Supplementary Document**). For non-normal distributions, the median was used for description, while for normal distributions by using the mean (standard deviation). Using ArcGIS 10.8 software to map the spatiotemporal distribution of HLE.

#### Spatial autocorrelation analysis

To establish the spatial relationship between HLE and health financing indicators, we used inverse distance spatial weights as a common metric between two units. We measured the distance between two areas and tested the spatial autocorrelation of HLE using the Moran’s I index of the exploratory spatial data analysis. We decomposed the results into global spatial autocorrelation analysis that used Moran’s results and the local spatial associations that used the Moran scatterplot of West Africa to inform local spatial associations.

#### Spatial panel data models

Based on results of the exploratory spatial data analysis, we used spatial panel data models to investigate the association between health financing and HLE at country level in West Africa during 1995-2019. We made a hypothesis maybe spatial spillover effects on the impact of health financing on HLE in long and short-terms. Specifically, spatial spillover effects include a direct impact on HLE from the unit itself, and an indirect impact on HLE of geographic proximity and eventually affect the unit in reverse [17]. We begin by selecting the appropriate models using the Lagrange multiplier test (LM test) and the robust test. We construct a spatial error model (SEM) and a spatial lag model (SLM) to take the spatial spillover effects into account in the data. Finally, we construct a spatial Durbin model (SDM) that combines both spatial lagged dependent and independent variables to provide further insights into the relationship between health financing and HLE. The SDM can be expressed as follows:

*y* = α + *u_i_* + *λ_t_* + *pWy* + *Xβ* + *WX̄γ* + ε

Where y is the dependent variable, representing the HLE;α refers to constant; *X* is an independent variable, representing indicators of health financing; *u_i_* represents spatial fixed effect; *λ_t_* represents time fixed effect; ρ refers to the estimated parameter; *W* represents the normalised spatial weight matrix; *X̄* represents matrix; γ refers to the parameter vector; and ε represents the random error.

The Wald test, Likelihood Ratio (LR) test, Akaike information criterion (AIC), Schwarz criterion (SC), log-likelihood, and R-squared were used to compare the goodness-of-fit degree of the two spatial regression models. Then we performed Hausman test to identify fixed or random effects of spatial effects and time effects to selected models. In addition, we constructed dynamic spatial Durbin model in our analysis, which SDM adding by time lagged dependent variable (DSDMlag1), SDM adding by space-time lagged dependent variable (DSDMlag2) and SDM adding by both space-time and time lagged dependent variable (DSDMlag3) in the regressors to investigate the time and the space-time effects of the association between health financing and HLE. Previous research inferred direct effects of measuring impacts of the change of independent variables on the dependent variables of the neighbourhood and indirect effects of measuring impacts of the change of independent variables on the dependent variables of the neighbourhood based on spatial Durbin model’s own partial derivative and cross-partial derivative [18]. Essentially, the direct effects represent local impacts and the indirect effect represent impacts on neighbours, which derive from the relationship of neighbouring health financing values to local HLE values [17]. Furthermore, we divided the effects estimated by DSDM models to long and short-terms direct/indirect effects to investigate the mechanism of how health financing transition influenced HLE in specific space-time specifications.

In this study, *P* < 0.05 was considered statistically significant and all tests were two sided. We conducted sensitivity analysis by changing the spatial weight matrix to a “Rook” matrix of adjacent points to explore association between health financing and HLE. All statistical analyses were performed by using Stata MP 17 (Stata Cooperation College Station, Texas, USA).

## RESULTS

### Disparities and spatial distribution HLE in West Africa, 1995-2019

**Figure 1:**
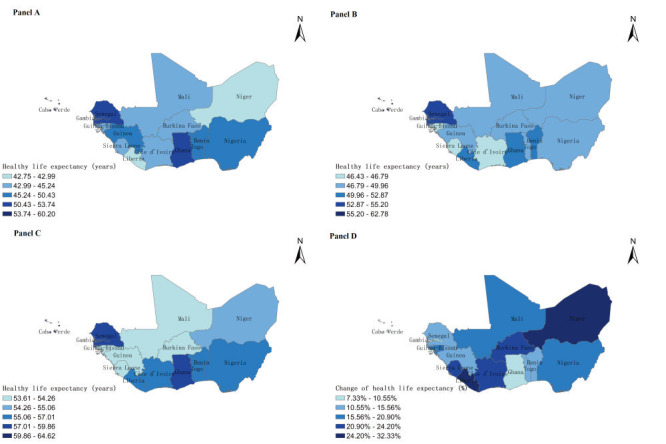
Spatial distribution of healthy life expectancy in West Africa. **Panel A.** 1995. **Panel B.** 2005. **Panel C.** 2019. **Panel D.** Change during 1995-2019.

**Figure 2:**
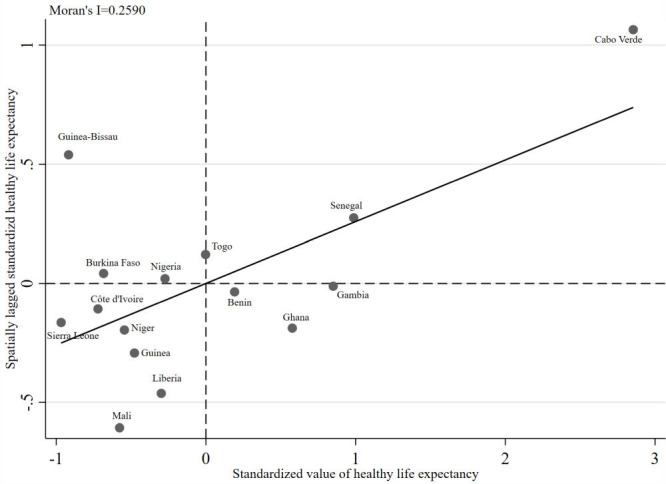
Moran scatterplot of mean healthy life expectancy in West Africa during 1995-2019.

The spatial distribution of HLE in West Africa in 1995, 2005, 2019 and change percentage of HLE between 1995 and 2019 were illustrated (**Figure 1**, panel A, panel B, panel C and panel D). Senegal and Ghana experienced the highest HLE in the study period in this region. HLE in Niger and Liberia experienced a trend of rapid growth since 1995. On the contrary, HLE in Ghana experienced change with the lowest percentage in this region. The Moran scatterplot was applied to describe spatial correlation of mean HLE during 1995-2019, which divided 15 countries into four quadrants by mean of HLE and spatially lagged HLE (**Figure 2**). These countries with mean HLE were high-high positive spatial correlation in the first quadrant, including Senegal with higher HLE was surrounded with similar higher HLE countries. Then, countries with mean HLE were low-high negative spatial correlation in the second quadrant, including Guinea-Bissau and Nigeria with lower HLE were surrounded with higher HLE countries. The third quadrant referred countries with mean HLE were low-low positive spatial correlation, such as Sierra Leone and Niger with lower HLE were surrounded with lower HLE countries. Lastly, the fourth quadrant referred countries with mean HLE were high-low spatial correlation, such as Benin with higher HLE was surrounded with lower HLE countries.

### Association between health financing and HLE in West Africa

Description of health financing variables for 15 west African countries was presented by mean (standard deviation (SD)) and median (25th percentile, Q1; 75th percentile, Q3) and was calculated on average for period 1995-1999, 2000-2014, 2015-2019 separately (**Table 1**). The statistical data revealed increasing trends of the indicators of health financing level and structure in the study period. Though overall level of total health spending, government health spending, out-of-pocket health spending and DAH were increased since 1995, government health spending per gross domestic product, prepaid private health spending per gross domestic product exhibited stable average in the three period. On the contrary, government health spending per person experienced decline trend from 1995 to 2019. Evidently, out-of-pocket health spending per person and out-of-pocket health spending per gross domestic product showed an incline trend on average since 1995. For structure of health financing, out-of-pocket health spending per total health spending was always the highest comparing other sources of health financing, which decreased from 57% during 1995-1999 to 42% during 2015-2019. DAH per total health spending increased from 11% during 1995-1999 to 22% during 2015-2019.

**Table 1:** Descriptive analysis of health financing on average of 15 countries in West Africa, 1995-2019

Health financing component		Variables	Description	Median (Q1*-Q3†)/Mean (SD)		
**1995-1999 on average**	**2000-2014 on average**	**2015-2019 on average**
Health financing level	Overall level	THS	Total Health Spending (2021, in USD)	190 000.00 (48 868.00-450 000.00)	310 000.00 (130 000.00-660 000.00)	600 000.00 (300 000.00-1 000 000.00)
		GHS	Government Health Spending	45 652.00 (18 922.00-130 000.00)	62 996.00 (24 651.00-180 000.00)	92 803.00 (49 004.00-310 000.00)
		PPHS	Prepaid Private Health Spending (2021, in USD)	9610.00 (1369.00-34 476.00)	11 678.00 (2721.00-51 276.00)	22 844.00 (9903.00-67 194.00)
		OOPHS	Out-of-pocket Health Spending (2021, in USD)	110 000.00 (25 606.00-250 000.00)	180 000.00 (54 623.00-310 000.00)	250 000.00 (120 000.00-480 000.00)
		DAH	Development Assistance for Health (2021, in USD)	25 577.00 (5661.00-38 397.00)	56 580.00 (27 673.00-140 000.00)	150 000(76 719-240 000)
	Per capita level	THS/PC	Total Health Spending per person (2021, in USD)	33.00 (25.00-40.00)	40.00 (30.00-60.00)	57.00 (41.00-71.00)
		GHS/PC	Government Health Spending per person (2021, in USD)	8.00 (5.00-11.00)	8.00 (6.00-13.00)	9.00 (7.00-17.00)
		PPHS/PC	Prepaid Private Health Spending per person (2021, in USD)	2.00 (1.00-2.00)	2.00 (1.00-2.00)	3.00 (1.00-4.00)
		OOPHS/PC	Out-of-pocket Health Spending per person (2021, in USD)	20.00 (14.00-25.00)	22.00 (14.00-28.00)	27.25 (13.29)
		DAH/PC	Development Assistance for Health per person (2021, in USD)	3.00 (1.00-4.00)	8.00 (5.00-12.00)	11.00 (9.00-22.00)
	Per GDP level	THS/GDP	Total Health Spending per Gross Domestic Product	0.04 (0.03-0.05)	0.05 (0.04-0.06)	0.05 (0.03-0.07)
		GHS/GDP	Government Health Spending per Gross Domestic Product	0.01 (0.01-0.01)	0.01 (0.01-0.01)	0.01 (0.01-0.01)
		PPHS/GDP	Prepaid Private Health Spending per Gross Domestic Product	0.00 (0.00-0.00)	0.00 (0.00-0.00)	0.00 (0.00-0.00)
		OOPHS/GDP	Out-of-pocket Health Spending per Gross Domestic Product	0.02 (0.02-0.03)	0.02 (0.01-0.03)	0.02 (0.01-0.03)
		DAH/GDP	Development Assistance for Health per Gross Domestic Product	0.00 (0.00-0.01)	0.01 (0.00-0.01)	0.01 (0.01-0.03)
Health financing structure	Government	GHS/THS	Government Health Spending per Total Health Spending	0.23 (0.16-0.31)	0.22 (0.14-0.31)	0.22 (0.14-0.31)
	Nongovernment	PPHS/THS	Prepaid Private Health Spending per Total Health Spending	0.06 (0.04)	0.05 (0.02-0.06)	0.05 (0.02-0.08)
		OOPHS/THS	Out-of-pocket Health Spending per Total Health Spending	0.59 (0.50-0.71)	0.50 (0.37-0.65)	0.44 (0.15)
	External assistance	DAH/THS	Development Assistance for Health per Total Health Spending	0.09 (0.05-0.14)	0.19 (0.13-0.28)	0.22 (0.16-0.34)

SD – standard deviation

*Q1 – 25th percentile.

†Q3 – 75th percentile.

The LM lag test, LM error test, Robust LM lag test, Robust LM error test, Wald test and LR test were used to diagnostics for spatial dependence to choose spatial panel data models from SEM, SAR and SDM. Both LM lag test (LM lag test statistics = 89.86, *P* < 0.001) and LM error test (LM error test statistics = 12.78, *P* < 0.001) statistics were significant, indicating spatial dependence in this case. Thus, the Robust LM test was applied for both SEM and SAR. The Robust LM lag test (Robust LM lag test statistics = 125.10, *P* < 0.001) and Robust LM error test (LM error test statistics = 48.01, *P* < 0.001) statistics were also significant, suggesting that the SDM is a better model in comparation with SEM and SAR. It revealed that SDM could not be simplified to SEM (Wald test statistics = 154.01, *P* < 0.001; LR test statistics = 124.26, *P* < 0.001) or SAR (Wald test statistics = 142.96, *P* < 0.001; LR test statistics = 119.66, *P* < 0.001). Hausman test (Hausman test statistics = 40.65, *P* < 0.01) proved that the rationality of using both spatial and time fixed effects model. The similar performance of DSDMlag1 and DSDMlag3 were shown in **Table 2** and **Table 3**, goodness-of-fit statistics such as AIC, BIC, R2 and Log-likelihood were applied to estimate the fitting degree of regressions. The AIC and BIC for DSDMlag3 were found higher than DSDMlag1, and values of R2 of DSDMlag1 were obviously higher than those of DSDMlag3, thus we selected DSDMlag1 in the following interpretation.

**Table 2:** The association between health financing and healthy life expectancy: spatial panel data models with main effects

Health financing component		Variables	Panel*	SDM	DSDMlag1	DSDMlag2	DSDMlag3
Health financing level	Overall level	THS	17.44 (8.01-26.87)†	-5.24 (-9.46,-1.01)†	-2.66 (-5.05,-0.26)†	-4.32 (-8.30,-0.35)†	-2.59 (-4.98,-0.20)†
		GHS	-7.01 (-11.40,-2.62)†	-1.91 (-3.75,-0.07)†	0.44 (-0.68,1.55)	-2.29 (-4.12,-0.46)†	0.50 (-0.62,1.62)
		PPHS	-0.67 (-2.64,1.30)	-1.73 (-2.50,-0.96)†	-0.16 (-0.62,0.29)	-1.56 (-2.29,-0.83)†	-0.18 (-0.63,0.27)
		OOPHS	-11.69 (-21.57,-1.81)†	20.32 (16.04-24.59)†	3.13 (0.41-5.85)†	18.74 (14.63-22.85)†	3.09 (0.38-5.81)†
		DAH	4.91 (2.88-6.94)†	-0.76 (-1.39,-0.13)†	0.24 (-0.17,0.64)	0.08 (-0.60,0.75)	0.24 (-0.17,0.65)
	Per capita level	THS/PC	-0.19 (-0.64,0.26)	-0.10 (-0.22,0.02)	-0.02 (-0.09,0.05)	-0.08 (-0.19,0.03)	-0.02 (-0.09,0.05)
		GHS/PC	0.37 (-0.09,0.82)	0.03 (-0.10,0.15)	0.00 (-0.08,0.07)	0.02 (-0.10,0.13)	0.00 (-0.07,0.07)
		PPHS/PC	-0.99 (-1.54,-0.44)†	0.06 (-0.13,0.25)	0.08 (-0.03,0.19)	0.13 (-0.05,0.31)	0.08 (-0.03,0.19)
		OOPHS/PC	0.26 (-0.19,0.71)	-0.01 (-0.13,0.11)	0.00 (-0.07,0.07)	-0.02 (-0.14,0.10)	0.00 (-0.06,0.07)
		DAH/PC	0.18 (-0.27,0.62)	0.12 (0.00-0.24)†	0.01 (-0.05,0.08)	0.10 (-0.01,0.21)	0.02 (-0.05,0.08)
	Per GDP level	THS/GDP	5.13 (-43.73,53.99)	-5.86 (-20.13,8.41)	5.49 (-2.95,13.94)	-9.81 (-23.88,4.26)	4.77 (-3.76,13.30)
		GHS/GDP	-2.09 (-89.01,84.83)	-17.28 (-43.33,8.77)	-3.57 (-18.19,11.05)	-10.22 (-34.48,14.05)	-3.30 (-17.9,11.31)
		PPHS/GDP	-61.97 (-216.16,92.23)	-29.20 (-84.47,26.07)	-24.18 (-55.75,7.40)	-51.27 (-103.72,1.17)	-25.65 (-57.27,5.97)
		OOPHS/GDP	-23.87 (-90.50,42.75)	-23.12 (-43.57,-2.68)†	2.14 (-9.79,14.07)	-29.08 (-48.54,-9.62)†	1.84 (-10.08,13.76)
		DAH/GDP	-54.56 (-122.32,13.20)	-18.86 (-37.28,-0.43)†	4.91 (-5.83,15.66)	-19.22 (-36.83,-1.61)	4.87 (-5.87,15.60)
Health financing structure	Government	GHS/THS	23.64 (-24.22,71.50)	9.57 (-3.23,22.37)	-0.08 (-7.28,7.13)	9.91 (-1.99,21.81)	-0.04 (-7.23,7.16)
	Nongovernment	PPHS/THS	113.57 (63.01-164.13)†	23.11 (7.91-38.32)†	-3.46 (-12.18,5.26)	20.24 (5.86-34.63)†	-2.79 (-11.60,6.02)
		OOPHS/THS	20.88 (-25.92,67.68)	-8.32 (-20.64,3.99)	-3.16 (-10.17,3.85)	-6.39 (-18.04,5.27)	-2.93 (-9.95,4.08)
	External assistance	DAH/THS	21.65 (-25.04,68.35)	5.72 (-6.69,18.14)	-1.77 (-8.75,5.22)	5.65 (-5.94,17.25)	-1.50 (-8.49,5.49)
Constant			2.91 (-43.37,49.20)	-	-	-	-
(t-1) × HLE			-	-	0.86 (0.80-0.92)		0.85 (0.79-0.91)
(t-1) w × HLE			-		-	-0.58 (-0.84,-0.31)	-0.09 (-0.27,0.10)

*Coefficient of ordinary panel data model.

†*P* < 0.05.

SDM – coefficient of spatial panel Durbin model, DSDMlag1 – coefficient of spatial panel Durbin mode with time lagged regressors, DSDMlag2 – coefficient of spatial panel Durbin mode with space- time lagged regressors, DSDMlag3 – coefficient of spatial panel Durbin mode with time and space-time lagged regressors, t – the study period 1995-2019, w – the spatial weight matrix at country level, THS – total health spending, GHS – government health spending, PPHS – prepaid private health spending, OOPHS – out-of-pocket health spending, DAH – development assistance for health, THS/PC – total health spending per person, GHS/PC – government health spending per person, PPHS/PC – prepaid private health spending per person, OOPHS/PC – out-of-pocket health spending per person, DAH/PC – development assistance for health per person, THS/GDP – total health spending per gross domestic product, GHS/GDP – government health spending per gross domestic product, PPHS/GDP – prepaid private health spending per gross domestic product, OOPHS/GDP – out-of-pocket health spending per gross domestic product, DAH/GDP – development assistance for health per gross domestic product, GHS/THS – government health spending per total health spending, PPHS/THS – prepaid private health spending per total health spending, OOPHS/THS – out-of-pocket health spending per total health spending, DAH/THS – development assistance for health per total health spending

**Table 3:** The association between health financing and healthy life expectancy: spatial panel data models with spatial lagged effects

Health financing component		Variables	Panel	SDM	DSDMlag1	DSDMlag2	DSDMlag3
Health financing level	Overall level	THS		8.14 (-2.17,18.46)	-1.43 (-7.34,4.48)	14.08 (4.18-23.98)*	-0.74 (-6.78,5.31)
		GHS		8.68 (4.19-13.17)*	2.96 (0.33-5.59)*	4.53 (0.10-8.95)*	2.71 (0.04-5.37)*
		PPHS		0.92 (-0.54,2.37)	0.11 (-0.74,0.96)	-0.11 (-1.53,1.30)	0.03 (-0.82,0.88)
		OOPHS		-16.28 (-26.12,-6.44)*	0.31 (-5.53,6.14)	-12.75 (-22.29,-3.20)*	0.46 (-5.37,6.28)
		DAH		0.70 (-0.57,1.97)	-0.79 (-1.57,-0.01)*	0.46 (-0.82,1.75)	-0.80 (-1.58,-0.02)*
	Per capita level	THS/PC		-0.29 (-0.53,-0.05)*	-0.01 (-0.15,0.13)	-0.33 (-0.56,-0.10)*	-0.02 (-0.16,0.12)
		GHS/PC		0.05 (-0.20,0.31)	-0.07 (-0.21,0.07)	0.06 (-0.18,0.30)	-0.06 (-0.21,0.08)
		PPHS/PC		0.49 (0.10-0.89)*	0.25 (0.02-0.48)*	0.65 (0.27-1.02)*	0.26 (0.03-0.49)*
		OOPHS/PC		0.41 (0.16-0.66)*	0.00 (-0.14,0.14)	0.43 (0.19-0.66)*	0.01 (-0.13,0.16)
		DAH/PC		0.24 (0.01-0.48)*	0.01 (-0.13,0.14)	0.29 (0.06-0.51)*	0.02 (-0.12,0.16)
	Per GDP level	THS/GDP		-0.26 (-28.27,27.75)	8.97 (-7.67,25.61)	-17.87 (-45.45,9.71)	8.14 (-8.55,24.84)
		GHS/GDP		30.36 (-14.22,74.93)	3.44 (-21.71,28.60)	28.75 (-12.89,70.40)	4.04 (-21.10,29.17)
		PPHS/GDP		-254.91 (-387.48,-122.34)*	-64.12 (-141.50,13.26)	-295.70 (-421.26,-170.14)*	-66.52 (-143.89,10.84)
		OOPHS/GDP		-23.32 (-60.88,14.24)	-3.77 (-25.14,17.60)	-32.90 (-68.87,3.07)	-6.08 (-27.80,15.64)
		DAH/GDP		-7.12 (-45.95,31.71)	-2.39 (-24.88,20.11)	-21.52 (-59.06,16.02)	-3.74 (-26.36,18.89)
Health financing structure	Government	GHS/THS		-8.62 (-31.22,13.99)	-1.21 (-14.01,11.59)	-1.74 (-23.01,19.54)	-0.93 (-13.73,11.87)
	Nongovernment	PPHS/THS		6.38 (-20.90,33.67)	-10.63 (-26.18,4.93)	13.77 (-12.16,39.70)	-9.46 (-25.15,6.22)
		OOPHS/THS		10.08 (-12.73,32.90)	0.61 (-12.60,13.82)	5.82 (-16.08,27.71)	0.34 (-12.86,13.53)
	External assistance	DAH/THS		-0.42 (-21.57,20.72)	0.23 (-11.70,12.16)	0.02 (-19.78,19.83)	0.16 (-11.75,12.08)
ρ			-	-0.28 (-0.43,-0.13)	0.03 (-0.09,0.15)	0.08 (-0.11,0.27)	0.01 (-0.15,0.17)
R^2^			0.75	0.08	0.93	0.11	0.89
AIC			1769.44	721.91	267.53	634.32	268.77
BIC			1844.05	878.99	426.86	793.65	431.99
Log-likelihood			-	-320.96	-582.99	-3351.75	-660.03

Panel – coefficient of ordinary panel data model, SDM – coefficient of spatial panel Durbin model, DSDMlag1 – coefficient of spatial panel Durbin mode with time lagged regressors, DSDMlag2 – coefficient of spatial panel Durbin mode with space- time lagged regressors, DSDMlag3 – coefficient of spatial panel Durbin mode with time and space-time lagged regressors, t – the study period 1995-2019, w – the spatial weight matrix at country level, THS – total health spending, GHS – government health Spending, PPHS – prepaid private health spending, OOPHS – out-of-pocket health spending, DAH – development assistance for health, THS/PC – total health spending per person, GHS/PC – government health spending per person, PPHS/PC – prepaid private health spending per person, OOPHS/PC – out-of-pocket health spending per person, DAH/PC – development assistance for health per person, THS/GDP – total health spending per gross domestic product, GHS/GDP – government health spending per gross domestic product, PPHS/GDP – prepaid private health spending per gross domestic product, OOPHS/GDP – out-of-pocket health spending per gross domestic product, DAH/GDP – development assistance for health per gross domestic product, GHS/THS – government health spending per total health spending, PPHS/THS – prepaid private health spending per total health spending, OOPHS/THS – out-of-pocket health spending per total health spending, DAH/THS – development assistance for health per total health spending, ρ – quantified the spatial autocorrelation magnitude of spatially lagged dependent variable, AIC – Akaike information criterion, BIC – Bayesian information criterion

*P < 0.05.

For the overall level of health financing in countries with lower total health spending (-2.66, 95% CI = -5.05,-0.26), higher out-of-pocket health spending (3.13, 95% CI = 0.41-5.85) was associated with an increase in HLE (**Table 2** and **Table 3**). For spatially lagged effects or neighbouring effects, government health spending (2.96, 95% CI = 0.33-5.59), DAH (-0.79, 95% CI = -1.57,-0.01) and prepaid private health spending per person (0.25, 95% CI = 0.02-0.48) exerted impacts on HLE among adjacent countries. Furthermore, we presented direct/indirect and long/short-term effects decomposition to interpret how health financing influence HLE variations based on DSDMlag1 estimation (**Table 4** and **Table 5**). For long-term effects, total health spending (-19.91, 95% CI = -38.15,-1.66) and out-of-pocket health spending (22.84, 95% CI = 3.47-42.22) statistically significant influenced HLE variations in direct. For details, the significant negative and positive correlation between total health spending and out-of-pocket health spending on HLE in a country over a long period of time. For short-term effects, it estimated that there were much more health financing variables presented significant influenced on HLE variations. total health spending (-2.7, 95% CI = -5.01,-0.39) and out-of-pocket health spending (3.13, 95% CI = 0.60-5.65) statistically significant influenced HLE variations in direct. Indicating that Total health spending and out-of-pocket health spending of a country in the previous year had significant negative and positive correlations with HLE of the country in the current year, respectively. government health spending (2.96, 95% CI = 0.34-5.57), DAH (-0.80, 95% CI = -1.57,-0.03) and prepaid private health spending per person (0.24, 95% CI = 0.02-0.46) statistically significant influenced HLE variations in indirect. It is argued that government health spending, DAH and prepaid private health spending per person in adjacent countries had positive and negative correlations with HLE respectively. Government health spending (3.44, 95% CI = 0.32-6.56) and prepaid private health spending per person (0.33, 95% CI = 0.07-0.58) statistically significant influenced HLE variations in total. In contrast with main effects, it was inferred that the short-term indirect effects occupied the dominant impacts of association between health financing and HLE variations. It was shown that DAH (-0.80, 95% CI = -1.57,-0.03) and prepaid private health spending per person (0.24, 95% CI = 0.02-0.46) were much different from that estimation shown in **Table 3**, which attributed to spatial spillover effects and main effects.

**Table 4:** Direct and Indirect effects decomposition during long-term of healthy life expectancy associated and health financing

Health financing component		Variables	Direct	Indirect	Total
Health financing level	Overall level	THS	-19.91 (-38.15,-1.66)*	-9.65 (-64.87,45.57)	-29.55 (-91.05,31.94)
		GHS	2.84 (-6.12,11.79)	21.08 (-7.28,49.45)	23.92 (-9.58,57.43)
		PPHS	-1.15 (-4.51,2.20)	1.17 (-5.81,8.15)	0.02 (-7.61,7.66)
		OOPHS	22.84 (3.47-42.22)*	-0.96 (-44.67,42.74)	21.88 (-25.98,69.74)
		DAH	2.01 (-1.26,5.28)	-5.89 (-12.37,0.60)	-3.88 (-11.77,4.00)
	Per capita level	THS/PC	-0.14 (-0.67,0.39)	-0.04 (-1.12,1.04)	-0.18 (-1.46,1.10)
		GHS/PC	-0.01 (-0.55,0.53)	-0.47 (-1.66,0.71)	-0.48 (-1.89,0.92)
		PPHS/PC	0.54 (-0.41,1.49)	1.66 (-0.52,3.84)	2.20(-0.40,4.80)
		OOPHS/PC	0.02 (-0.52,0.56)	0.01 (-1.10,1.12)	0.03 (-1.27,1.32)
		DAH/PC	0.10 (-0.44,0.63)	0.06 (-1.01,1.13)	0.16 (-1.12,1.44)
	Per GDP level	THS/GDP	40.36 (-31.23,111.95)	57.67 (-105.68,221.02)	98.04 (-90.59,286.67)
		GHS/GDP	-32.12 (-139.68,75.44)	22.17 (-170.10,214.45)	-9.95 (-236.01,216.12)
		PPHS/GDP	-154.87 (-405.23,95.49)	-418.00 (-1055.55,219.55)	-572.87 (-1353.48,207.74)
		OOPHS/GDP	16.02 (-74.96,106.99)	-20.08 (-185.98,145.83)	-4.06 (-193.76,185.63)
		DAH/GDP	38.87 (-41.35,119.09)	-20.73 (-183.75,142.29)	18.14 (-161.71,198.00)
Health financing structure	Government	GHS/THS	2.17 (-52.69,57.03)	-8.77 (-116.36,98.82)	-6.60 (-134.89,121.69)
	Nongovernment	PPHS/THS	-21.44 (-91.80,48.92)	-76.86 (-234.03,80.32)	-98.30 (-289.33,92.74)
		OOPHS/THS	-19.90 (-73.65,33.86)	7.24 (-94.93,109.42)	-12.65 (-134.62,109.32)
	External assistance	DAH/THS	-10.05 (-63.42,43.33)	2.84 (-95.52,101.20)	-7.21 (-127.62,113.20)

Direct – coefficient of direct effects, Indirect – coefficient of indirect effects, Total – coefficient of total effects, THS – total health spending, GHS – government health spending, PPHS – prepaid private health spending, OOPHS – out-of-pocket health spending, DAH – development assistance for health, THS/PC – total health spending per person, GHS/PC – government health spending per person, PPHS/PC – prepaid private Health Spending per person, OOPHS/PC – Out-of-pocket Health Spending per person, DAH/PC – Development Assistance for Health per person, THS/GDP – total health spending per gross domestic product, GHS/GDP – government health spending per gross domestic product, PPHS/GDP – prepaid private health spending per gross domestic product, OOPHS/GDP – out-of-pocket health spending per gross domestic product, DAH/GDP – development assistance for health per gross domestic product, GHS/THS – government health spending per total health spending, PPHS/THS – prepaid private health spending per total health spending, OOPHS/THS – out-of-pocket health spending per total health spending, DAH/THS – development assistance for health per total health spending

*P < 0.05.

**Table 5:** Direct and Indirect effects decomposition during short-term of healthy life expectancy associated and health financing

Health financing component		Variables	Direct	Indirect	Total
Health financing level	Overall level	THS	-2.70 (-5.01,-0.39)*	-1.38 (-7.04,4.27)	-4.08 (-10.54,2.38)
		GHS	0.48 (-0.58,1.55)	2.96 (0.34,5.57)*	3.44 (0.32-6.56)*
		PPHS	-0.17 (-0.60,0.27)	0.11 (-0.74,0.97)	-0.05 (-0.99,0.89)
		OOPHS	3.13 (0.60-5.65)*	0.21 (-5.21,5.63)	3.34 (-2.89,9.57)
		DAH	0.24 (-0.15,0.63)	-0.80 (-1.57,-0.03)*	-0.56 (-1.50,0.37)
	Per capita level	THS/PC	-0.02 (-0.09,0.05)	-0.01 (-0.15,0.13)	-0.03 (-0.20,0.14)
		GHS/PC	0.00 (-0.07,0.07)	-0.07 (-0.21,0.08)	-0.07 (-0.25,0.11)
		PPHS/PC	0.08 (-0.03,0.20)	0.24 (0.02-0.46)*	0.33 (0.07-0.58)*
		OOPHS/PC	0.00 (-0.07,0.07)	0.00 (-0.14,0.14)	0.01 (-0.16,0.18)
		DAH/PC	0.01 (-0.06,0.08)	0.01 (-0.12,0.15)	0.02 (-0.14,0.19)
	Per GDP level	THS/GDP	5.67 (-3.37,14.71)	7.86 (-8.72,24.45)	13.54 (-5.67,32.74)
		GHS/GDP	-4.15 (-18.45,10.15)	2.81 (-21.69,27.31)	-1.33 (-31.46,28.80)
		PPHS/GDP	-23.91 (-56.73,8.91)	-62.64 (-135.09,9.82)	-86.55(-178.64,5.55)
		OOPHS/GDP	1.87 (-10.17,13.92)	-3.24 (-23.98,17.50)	-1.37 (-25.97,23.24)
		DAH/GDP	5.10 (-5.49,15.69)	-2.69 (-23.97,18.59)	2.41 (-21.90,26.71)
Health financing structure	Government	GHS/THS	0.26 (-6.88,7.40)	-1.16 (-13.74,11.42)	-0.90 (-16.27,14.47)
	Nongovernment	PPHS/THS	-3.20 (-12.04,5.64)	-10.51 (-25.65,4.63)	-13.71 (-32.98,5.55)
		OOPHS/THS	-2.73 (-9.83,4.37)	0.75 (-11.94,13.44)	-1.98 (-17.59,13.64)
	External assistance	DAH/THS	-1.36 (-8.38,5.65)	0.34 (-11.41,12.09)	-1.02 (-15.89,13.84)

Direct – coefficient of direct effects, Indirect – coefficient of indirect effects, Total – coefficient of total effects, THS – total health spending, GHS – government health spending, PPHS – prepaid private health spending, OOPHS – out-of-pocket health spending, DAH – development assistance for health, THS/PC – total health spending per person, GHS/PC – government health spending per person, PPHS/PC – prepaid private health spending per person, OOPHS/PC – out-of-pocket health spending per person, DAH/PC – development assistance for health per person, THS/GDP – total health spending per gross domestic product, GHS/GDP – government health spending per gross domestic product, PPHS/GDP – prepaid private health spending per gross domestic product, OOPHS/GDP – out-of-pocket health spending per gross domestic product, DAH/GDP – development assistance for health per gross domestic product, GHS/THS – government health spending per total health spending, PPHS/THS – prepaid private health spending per total health spending, OOPHS/THS – out-of-pocket health spending per total health spending, DAH/THS – development assistance for health per total health spending

*P < 0.05.

## DISCUSSION

Spatial variations of HLE existed at country-level in West Africa. From a spatial perspective, our hypothesis was accepted and the dynamic analysis revealed that there were spatial spillover effects, direct effects, and indirect effects between health financing structure and level and HLE both the long-term and the short-term. Total health spending and out-of-pocket health spending affected HLE positively and negatively in the long-term, respectively. The positive effects to local country and adjacent country HLE from government health spending and prepaid private health spending per person, and negative effects to local country and adjacent country HLE from DAH in the short-term. In particular, the short-term spatial spillover effects of government health spending, DAH, and prepaid private health spending per person were more pronounced than the long-term effects.

The HLE in West Africa has been slowly declining compared to the rest of the world, which may be closely related to weak health system and economic development. Existing spatial autocorrelation in HLE and adjacent countries exerted spatial effects on the other. Consistent with previous cross-national studies have also described the spatiotemporal trends of life expectancy, and revealed African countries experienced the lowest life expectancies [19]. Additionally, spatial dependence has also been explained in both developed countries [20-23] (such as the UK, South Korea, and the US) and developing countries (such as China) [12,24]. Although development of economy maintained homologous in West Africa, it is argued that existed disparities in HLE among countries, highlighting the enormous challenge of addressing health inequality in the region.

Our findings indicated that the main effects and spatial spillover effects showed that total health spending and out-of-pocket health spending were associated with HLE, as well as government health spending, DAH and prepaid private health spending per person were associated with neighbourhood HLE. Contrary to our study, previous research using spatial Durbin panel model showed positive effects between total health spending and life expectancy at the province level in China [12].This may be due to China’s reasonable allocation of health financing at the administrative area level, while each West African country planned its health resources and spending independently. Previous limited study analysed positive correlation between public health spending and out-of-pocket health spending with health outcomes in sub-Saharan African countries from a non-spatial perspective, revealed a stronger impact of public health spending [6]. Specifically, previous research also showed that a 1% increase in government health spending would increase in life expectancy by 6%, while a 1% increase in out-of-pocket health spending would increase in life expectancy by 63% in Nigeria [25]. The structure of health financing has primarily been composed of out-of-pocket health spending, followed by government health spending, and proportion of DAH has gradually increased in West African countries [14]. The HLE of a country is positively influenced by prepaid private health spending in adjacent countries in this study. Ghana introduced national health insurance in 2004, but out-of-pocket health spending still accounted for the largest proportion of Ghana's total health spending until 2017 [26]. Compared with government health financing mechanisms, prepaid private health spending (such as social health insurance) may require higher costs in the long-term, while government sources of health financing may require lower costs in the short-term [5]. Significant investments in technology, management and supply systems could needed to manage the prepaid private health spending. However, social health insurance systems still develop differently in West Africa, with some countries not implementing such systems.

We performed DSDM to interpret long and short-terms direct/indirect effects between health financing and HLE. Although previous research using SDM has explained association between health spending and health outcomes, gap existed in research for long and short-terms from a spatial perspective [12]. The positive direct effect was observed between both total health spending and out-of-pocket health spending and HLE during the long-term of 1995-2019 in our study. It is argued that Total health spending and out-of-pocket health spending affected HLE in local country in the long-term, spatial spillover effects may be covert. Previous limited research showed the long-term impacts between health spending and life expectancy in China from a non-spatial perspective [8]. Moreover, positive long-term effects between health spending and life expectancy was observed in Eastern Europe [27]. government health spending increased rapidly after MDGs were proposed in Sub-Saharan African countries, especially during 2000-2015 [28]. DAH also supported health system to decline health disparities in West Africa in the long-term. The effects of DAH provide information for evaluating of the recipient country. Since 1995, while significant increase in DAH was represented in West Africa [29], more effective spending on health is needed in most West African countries. In addition, it is important to consider the long-term impacts of global financial crisis [16,30].

Evidently, it was argued that health spending might improve HLE both in long and short-terms, which short-term spatial effects were more obvious in adjacent countries. On the one hand, increasing total health spending and out-of-pocket health spending might be related to improving HLE in the following year. On the other hand, out-of-pocket health spending accounted for structure of health financing and made great progress in improving health outcomes. HLE in a country in the present year was influenced by government health spending, DAH and prepaid private health spending per person of adjacent countries in the previous year. Moreover, HLE in adjacent countries also affected HLE of the country. In the short term, spatial spillover effect was more evident, which may be masked by impact of economy and other social determinants of health. However, it was taken more seriously factors may have significant impact on HLE in the long-term. For details, previous research explained social determinants of health, climate and economy were associated with life expectancy from a spatial perspective [19,31]. In terms of prepaid private health spending, social health insurance was scarce in West African countries, which might exert influences on local HLE from adjacent countries. Historically, health DAH focus areas have not aligned closely with disease burden [32] affecting HLE. If epidemic spreads in adjacent countries due to geographic proximity and population mobility, it can affect the population health of adjacent countries [33]. Additionally, government health spending per person and prepaid private health spending per person were also exerted influences on adjacent countries when population structure was considered. Consistent with previous studies, the GDP of a country was a significant factor on government health spending [34,35]. However, there was no significant association between government health spending per gross domestic product and HLE in both long-term and short-term. As the proportion of DAH to total health spending has gradually increased, increasing dependence on DAH and excessive expectations of DAH were represented in West Africa, which may also weaken the functionality of health financing. Therefore, the long-term and short-term spatial effects of health financing should be considered by health policymakers and regional organisations when formulating health policies.

Few studies have attempted to explore the long-term and short-term effects of the transition of health financing structure in West Africa on HLE from a spatial perspective. Through introducing ecological model of health financing, our study made full use of spatial panel data when interpreting the time varying change of health financing and HLE disparities. However, this study was also subjected to several limitations. First, it is an ecological study that explored statistical correlation between health financing and HLE from a spatial perspective using panel data, thus it cannot reveal causal relationships. Second, the data has been estimated based on statistical models, and the weak health systems and insufficient health data in West African countries have influenced the quality of result to some extent. Third, indicators of health financing could be further refined. On the hand, public health spending has profound impact on improving population health [36], but this study excluded this indicator. On the other hand, development assistance for health has not been refined to key areas of assistance. So can analyse the spatial effects of DAH on health of West Africa from HIV/AIDS, malaria, tuberculosis, non-communicable diseases, and maternal and child health in the future [29,30,32]. Fourth, data quality and model parameters might lead to uncertainties of results estimations, hence omitted variables and potential confounders were inevitable like national culture, macrolevel events (such as Ebola), environmental metrics and national legislation. Fifth, the analysis done at country level but assumed homogeneity within one country, obtain county-level data to analyse the relationship between health financing and HLE in future research.

## CONCLUSIONS

In conclusion, our study demonstrated that disparities and spatial distribution of HLE exist at the country level in West Africa. Health financing regarding government, non-government, as well as external assistance not only affected HLE disparities at local scale but also among adjacent countries. Therefore, policy makers and health advocates must consider adjacent countries both long and short-term in health financing transition to ensure rational health financing resources for health in West Africa. By doing so can make significant progress in global health governance and reduce health inequalities in the region.

## Additional material


Online Supplementary Document
