# Supplementary material for: Spatiotemporal patterns of healthy life expectancy and the effects of health financing in West African countries, 1995-2019: A Spatial Panel Modelling Study
Source: J Glob Health. 2023 Oct 20;13:04123. doi: 10.7189/jogh.13.04123 (PMC10588290; doi:10.7189/jogh.13.04123)
Supplement: Online Supplementary Document [file jogh-13-04123-s001.pdf]

Supplemental Documents

Table S1. Normal test of health financing indicators.

Variable	1995-1999				2000-2014				2015-2019			
	P(skewness)	P(kurtosis)	Joint test		*P*(skewness)	*P*(kurtosis)	Joint test		*P*(skewness)	*P*(kurtosis)	Joint test	
			Adj chi2(2)	*P*			Adj chi2(2)	*P*			Adj chi2(2)	*P*
THS	<0.001	<0.001	47.16	<0.001	<0.001	<0.001	141.64	<0.001	<0.001	<0.001	52.11	<0.001
GHS	<0.001	<0.001	49.31	<0.001	<0.001	<0.001	115.45	<0.001	<0.001	<0.001	41.00	<0.001
PPHS	<0.001	0.0016	29.69	<0.001	<0.001	<0.001	73.77	<0.001	<0.001	0.0019	27.82	<0.001
OOPHS	<0.001	<0.001	48.22	<0.001	<0.001	<0.001	146.95	<0.001	<0.001	<0.001	53.94	<0.001
DAH	<0.001	0.0057	19.58	0.0001	<0.001	<0.001	160.04	<0.001	<0.001	<0.001	46.04	<0.001
THS/PC	<0.001	0.0199	18.97	0.0001	<0.001	<0.001	89.80	<0.001	<0.001	<0.001	41.46	<0.001
GHS/PC	<0.001	<0.001	51.22	<0.001	<0.001	<0.001	116.72	<0.001	<0.001	<0.001	50.74	<0.001
PPHS/PC	<0.001	<0.001	42.34	<0.001	<0.001	<0.001	84.71	<0.001	<0.001	0.0002	32.16	<0.001
OOPHS/PC	<0.001	0.0025	20.05	<0.001	<0.001	<0.001	48.18	<0.001	0.2647	0.0890	4.26	0.1186
DAH/PC	<0.001	<0.001	65.80	<0.001	<0.001	<0.001	114.40	<0.001	<0.001	<0.001	47.43	<0.001
THS/GDP	<0.001	0.0024	24.60	<0.001	<0.001	<0.001	86.49	<0.001	<0.001	<0.001	45.75	<0.001
GHS/GDP	<0.001	<0.001	37.21	<0.001	<0.001	0.0188	33.30	<0.001	<0.001	0.0057	22.19	<0.001
PPHS/GDP	0.0025	0.0583	10.67	0.0048	<0.001	0.0057	32.97	<0.001	0.1484	0.0038	9.04	0.0109
OOPHS/GDP	<0.001	0.0012	26.83	<0.001	<0.001	<0.001	75.55	<0.001	0.0001	0.0599	15.13	0.0005
DAH/GDP	<0.001	<0.001	72.37	<0.001	<0.001	<0.001	156.51	<0.001	<0.001	<0.001	59.53	<0.001
GHS/THS	<0.001	0.0001	33.96	<0.001	<0.001	0.0037	32.53	<0.001	0.0097	0.5541	6.52	0.0385
PPHS/THS	0.0711	0.6034	3.67	0.1599	<0.001	0.0029	32.89	<0.001	<0.001	0.0067	21.55	<0.001
OOPHS/THS	0.0036	0.2689	8.50	0.0143	0.4169	<0.001	30.21	<0.001	0.6514	0.9605	0.21	0.9019
DAH/THS	<0.001	0.0001	30.88	<0.001	<0.001	0.0020	38.15	<0.001	0.0001	0.0381	15.45	0.0004

Adj chi2(2) – chi-square value; THS – Total Health Spending; GHS – Government Health Spending; PPHS – Prepaid Private Health Spending; OOPHS – Out-of-pocket Health Spending; DAH – Development Assistance for Health; THS/PC – Total Health Spending per person; GHS/PC – Government Health Spending per person; PPHS/PC – Prepaid Private Health Spending per person; OOPHS/PC – Out-of-pocket Health Spending per person; DAH/PC – Development Assistance for Health per person; THS/GDP – Total Health Spending per Gross Domestic Product; GHS/GDP – Government Health Spending per Gross Domestic Product; PPHS/GDP – Prepaid Private Health Spending per Gross

Domestic Product; OOPHS/GDP – Out-of-pocket Health Spending per Gross Domestic Product; DAH/GDP – Development Assistance for Health per Gross Domestic Product; GHS/THS – Government Health Spending per Total Health Spending; PPHS/THS – Prepaid Private Health Spending per Total Health Spending; OOPHS/THS – Out-of-pocket Health Spending per Total Health Spending; DAH/THS – Development Assistance for Health per Total Health Spending.

Table S2. The association between health financing and HLE in West Africa, 1995-2019: estimated from spatial panel data models with main effects by using different spatial weight matrix.

Health financing component		Variables	Panel	SDM	DSDMlag1	DSDMlag2	DSDMlag3
Health financing level	Overall level	THS	-10.71(-22.09,0.66)	0.6(-4.78,5.98)	-3.7(-6.98,-0.42)‡	3.65(-1.43,8.73)	-3.58(-6.91,-0.25)‡
		GHS	7.8(2.37,13.22)‡	-1.68(-3.61,0.24)	0.42(-0.79,1.63)	-2.21(-4.04,-0.38)‡	0.42(-0.78,1.62)
		PPHS	-1.1(-3.03,0.83)	-1.79(-2.55,-1.02)‡	-0.17(-0.64,0.31)	-1.43(-2.14,-0.71)‡	-0.17(-0.64,0.3)
		OOPHS	2.02(-8.08,12.12)	18.39(13.44,23.34)‡	3.88(0.67,7.09)‡	14.8(10.04,19.56)‡	3.86(0.66,7.06)‡
		DAH	4.21(2.22,6.19)‡	-0.88(-1.52,-0.24)‡	0.33(-0.12,0.78)	0.14(-0.56,0.84)	0.33(-0.12,0.78)
	Per capita level	THS/PC	-0.12(-0.55,0.31)	-0.18(-0.3,-0.06)‡	-0.03(-0.1,0.04)	-0.17(-0.28,-0.06)‡	-0.03(-0.1,0.04)
		GHS/PC	0.49(0.04,0.95)‡	0.02(-0.11,0.15)	0.03(-0.05,0.1)	0(-0.12,0.12)	0.02(-0.05,0.1)
		PPHS/PC	-1.33(-1.87,-0.78)‡	0.19(-0.01,0.39)	0.1(-0.02,0.22)	0.28(0.1,0.46)‡	0.1(-0.02,0.22)
		OOPHS/PC	0.18(-0.25,0.61)	0.04(-0.08,0.16)	0.02(-0.06,0.09)	0.03(-0.08,0.15)	0.02(-0.06,0.09)
		DAH/PC	0.35(-0.08,0.77)	0.13(0.01,0.25)‡	0.02(-0.05,0.09)	0.11(0,0.22)	0.02(-0.05,0.09)
	Per GDP level	THS/GDP	-29.01(-76.65,18.62)	2.39(-12.07,16.85)	4.88(-4.13,13.89)	-2.04(-16.01,11.93)	4.68(-4.35,13.7)
		GHS/GDP	-97.65(-183.76,-11.55)‡	-12.25(-37.29,12.79)	1.08(-13.69,15.84)	-4.07(-26.87,18.73)	1.19(-13.52,15.9)
		PPHS/GDP	-43.57(-203.72,116.58)	-12.5(-66.44,41.44)	-21.08(-53.64,11.47)	-29.47(-79.73,20.8)	-21.45(-53.88,10.99)
		OOPHS/GDP	29.98(-35.24,95.2)	-19.48(-39.66,0.69)	-1.46(-13.77,10.85)	-25.27(-44.03,-6.51)‡	-1.57(-13.83,10.69)
		DAH/GDP	-116.86(-187.01,-46.72)‡	0.41(-19.47,20.29)	11.5(-0.52,23.53)	0.2(-18.31,18.7)	11.52(-0.45,23.49)
Health financing structure	Government	GHS/THS	-0.94(-47.59,45.72)	19.36(6.65,32.06)‡	-1.12(-8.8,6.56)	20.79(9.17,32.41)‡	-0.95(-8.65,6.74)
	Nongovernment	PPHS/THS	137.57(87.73,187.41)‡	24.7(9.26,40.15)‡	-4.17(-13.55,5.2)	20.13(5.86,34.4)‡	-3.97(-13.38,5.44)
		OOPHS/THS	21.73(-23.15,66.61)	0.18(-11.92,12.28)	-3.46(-10.78,3.85)	4.16(-7.18,15.5)	-3.3(-10.64,4.03)
	External assistance	DAH/THS	34.03(-10.93,78.99)	12.39(0.23,24.55)‡	-1.75(-9.04,5.53)	12.33(1.15,23.51)‡	-1.6(-8.9,5.69)
Constant			20.85(-23.97,65.68)	-	-	-	-

(t-1) × HLE	-	-	0.87(0.8,0.93)	-	0.86(0.79,0.93)
(t-1)w × HLE	-	0.28(0.24,0.32)	-	-0.76(-1.03,-0.5)	-0.03(-0.22,0.17)

Panel – ordinary panel data model; SDM – spatial panel Durbin model; DSDMlag1 – spatial panel Durbin mode with time lagged regressors; DSDMlag2 – spatial panel Durbin mode with space- time lagged regressors; DSDMlag3 – spatial panel Durbin mode with time and space-time lagged regressors; t – the study period 1995-2019; w – the spatial weight matrix at country level; ‡*P*<0.05; THS – Total Health Spending; GHS – Government Health Spending; PPHS – Prepaid Private Health Spending; OOPHS – Out-of-pocket Health Spending; DAH – Development Assistance for Health; THS/PC – Total Health Spending per person; GHS/PC – Government Health Spending per person; PPHS/PC – Prepaid Private Health Spending per person; OOPHS/PC – Out-of-pocket Health Spending per person; DAH/PC – Development Assistance for Health per person; THS/GDP – Total Health Spending per Gross Domestic Product; GHS/GDP – Government Health Spending per Gross Domestic Product; PPHS/GDP – Prepaid Private Health Spending per Gross Domestic Product; OOPHS/GDP – Out-of-pocket Health Spending per Gross Domestic Product; DAH/GDP – Development Assistance for Health per Gross Domestic Product; GHS/THS – Government Health Spending per Total Health Spending; PPHS/THS – Prepaid Private Health Spending per Total Health Spending; OOPHS/THS – Out-of-pocket Health Spending per Total Health Spending; DAH/THS – Development Assistance for Health per Total Health Spending.

Table S3. The association between health financing and HLE in West Africa, 1995-2019: estimated from spatial panel data models with spatial lagged effects by using different spatial weight matrix.

**Health financing component**		**Variables**	**Panel**	**SDM**	**DSDMlag1**	**DSDMlag2**	**DSDMlag3**
Health financing level	Overall level	THS		15.79(4.06,27.53)‡	-6.87(-14.21,0.48)	23.4(12.32,34.49)‡	-6.52(-14.06,1.03)
		GHS		9.37(4.73,14.02)‡	3.42(0.56,6.28)‡	5.21(0.77,9.66)‡	3.36(0.48,6.23)‡
		PPHS		1.31(-0.14,2.76)	0.19(-0.72,1.09)	0.18(-1.22,1.57)	0.16(-0.74,1.06)
		OOPHS		-14.78(-25.7,-3.86)‡	6.12(-0.84,13.07)	-9.97(-20.49,0.55)	6.16(-0.77,13.08)
		DAH		0.08(-1.19,1.35)	-0.99(-1.81,-0.16)‡	-0.18(-1.45,1.09)	-0.99(-1.81,-0.17)‡
	Per capita level	THS/PC		-0.39(-0.66,-0.13)‡	-0.01(-0.17,0.15)	-0.43(-0.68,-0.18)‡	-0.01(-0.18,0.15)
		GHS/PC		-0.08(-0.36,0.2)	-0.02(-0.19,0.15)	-0.1(-0.36,0.16)	-0.02(-0.18,0.15)
		PPHS/PC		0.56(0.13,0.98)‡	0.31(0.05,0.56)‡	0.7(0.3,1.09)‡	0.31(0.06,0.57)‡
		OOPHS/PC		0.47(0.2,0.75)‡	-0.02(-0.19,0.15)	0.47(0.22,0.73)‡	-0.02(-0.19,0.15)
		DAH/PC		0.26(0,0.52)	0.01(-0.14,0.17)	0.28(0.04,0.53)‡	0.02(-0.14,0.17)
	Per GDP level	THS/GDP		-5.72(-35.92,24.48)	1.59(-16.84,20.03)	-28.36(-56.73,0.02)	1.24(-17.21,19.69)
		GHS/GDP		71.65(23.59,119.7)‡	13.6(-15.07,42.28)	70.04(26.29,113.79)‡	14.02(-14.57,42.6)

		PPHS/GDP		-191.86(-323.44,-60.28)‡	-48.81(-129.46,31.84)	-211.37(-334.08,-88.66)‡	-49.58(-129.88,30.71)
		OOPHS/GDP		-20.23(-57.41,16.95)	-11.73(-34,10.54)	-37.43(-72.42,-2.44)‡	-12.66(-35.31,9.99)
		DAH/GDP		24.27(-19.77,68.31)	10.8(-15.85,37.44)	14.57(-26.69,55.84)	10.48(-16.14,37.1)
Health financing structure	Government	GHS/THS		5.61(-18.01,29.24)	-2.92(-17.03,11.19)	15.95(-5.86,37.75)	-2.6(-16.74,11.53)
	Nongovernment	PPHS/THS		1.65(-27.35,30.65)	-17.18(-34.72,0.35)	11.08(-16.09,38.24)	-16.67(-34.32,0.99)
		OOPHS/THS		13.95(-9.45,37.35)	0.3(-13.96,14.56)	12.24(-9.72,34.19)	0.31(-13.89,14.5)
	External assistance	DAH/THS		6.56(-16.09,29.2)	2.36(-11.05,15.77)	9.93(-10.77,30.64)	2.48(-10.88,15.84)
ρ			-	-0.37(-0.52,-0.21)	0.02(-0.1,0.15)	0.09(-0.1,0.28)	0.01(-0.15,0.18)
R^2			0.64	0.95	0.98	0.93	0.98
AIC			1603.17	643.24	247.66	543.43	249.24
BIC			1680.33	797.56	404.16	699.93	409.56
Log-likelihood			-	-281.62	-308.95	-1145.34	-321.62

Panel – ordinary panel data model; SDM – spatial panel Durbin model; DSDMlag1 – spatial panel Durbin mode with time lagged regressors; DSDMlag2 – spatial panel Durbin mode with space- time lagged regressors; DSDMlag3 – spatial panel Durbin mode with time and space-time lagged regressors; t – the study period 1995-2019; w – the spatial weight matrix at country level; ‡$P<0.05$; THS – Total Health Spending; GHS – Government Health Spending; PPHS – Prepaid Private Health Spending; OOPHS – Out-of-pocket Health Spending; DAH – Development Assistance for Health; THS/PC – Total Health Spending per person; GHS/PC – Government Health Spending per person; PPHS/PC – Prepaid Private Health Spending per person; OOPHS/PC – Out-of-pocket Health Spending per person; DAH/PC – Development Assistance for Health per person; THS/GDP – Total Health Spending per Gross Domestic Product; GHS/GDP – Government Health Spending per Gross Domestic Product; PPHS/GDP – Prepaid Private Health Spending per Gross Domestic Product; OOPHS/GDP – Out-of-pocket Health Spending per Gross Domestic Product; DAH/GDP – Development Assistance for Health per Gross Domestic Product; GHS/THS – Government Health Spending per Total Health Spending; PPHS/THS – Prepaid Private Health Spending per Total Health Spending; OOPHS/THS – Out-of-pocket Health Spending per Total Health Spending; DAH/THS – Development Assistance for Health per Total Health Spending.

Table S4. Direct and Indirect effects decomposition during long-term of HLE associated and health financing by using different spatial weight matrix in West Africa, 1995-2019.

Health financing component		Variables	Direct	Indirect	Total
Health financing level	Overall level	THS	-28.75(-106.19,48.69)	-50.01(-984.05,884.03)	-78.76(-1081.8,924.27)
		GHS	3.11(-18.5,24.72)	27.4(-184.84,239.64)	30.51(-198.64,259.66)
		PPHS	-1.22(-5.68,3.25)	1.77(-20.05,23.6)	0.56(-23.4,24.52)
		OOPHS	29.38(-14.04,72.8)	49.84(-310.02,409.7)	79.22(-311.07,469.51)
		DAH	2.93(-3.79,9.64)	-8.35(-41.31,24.62)	-5.42(-41.18,30.34)
	Per capita level	THS/PC	-0.21(-1.11,0.68)	0.18(-8.64,8.99)	-0.03(-9.51,9.44)
		GHS/PC	0.21(-0.48,0.9)	-0.15(-3.18,2.89)	0.06(-3.35,3.47)
		PPHS/PC	0.69(-1.89,3.26)	1.94(-30.11,34)	2.63(-31.71,36.97)
		OOPHS/PC	0.1(-0.86,1.06)	-0.45(-10.55,9.65)	-0.35(-11.19,10.49)
		DAH/PC	0.13(-0.67,0.92)	-0.04(-6.7,6.61)	0.09(-7.1,7.27)
	Per GDP level	THS/GDP	39.04(-144.6,222.68)	-36.58(-2385.51,2312.36)	2.46(-2512.36,2517.28)
		GHS/GDP	2.48(-134.79,139.74)	125.48(-387.42,638.39)	127.96(-452.09,708.01)
		PPHS/GDP	-152.51(-560.38,255.36)	-323.12(-4514.13,3867.89)	-475.63(-4982.52,4031.26)
		OOPHS/GDP	-9.44(-126.47,107.6)	-94.62(-384.41,195.16)	-104.06(-436,227.87)
		DAH/GDP	91.07(-86.08,268.22)	61.78(-1834.78,1958.33)	152.85(-1882.75,2188.45)
Health financing structure	Government	GHS/THS	-6.78(-79.46,65.9)	-34.06(-279.34,211.22)	-40.84(-328.05,246.37)
	Nongovernment	PPHS/THS	-28.68(-184.52,127.16)	-125.99(-1884.48,1632.5)	-154.67(-2044.8,1735.46)
		OOPHS/THS	-23.37(-100.25,53.5)	12.92(-612.52,638.37)	-10.45(-686.14,665.23)
	External assistance	DAH/THS	-11.46(-81.01,58.08)	24.54(-312.45,361.53)	13.08(-361.69,387.85)

‡$P<0.05$; THS – Total Health Spending; GHS – Government Health Spending; PPHS – Prepaid Private Health Spending; OOPHS – Out-of-pocket Health Spending; DAH – Development Assistance for Health; THS/PC – Total Health Spending per person; GHS/PC – Government Health Spending per person; PPHS/PC – Prepaid Private Health Spending per person; OOPHS/PC – Out-of-pocket Health Spending per person; DAH/PC – Development Assistance for Health per person; THS/GDP – Total Health Spending per Gross Domestic Product; GHS/GDP – Government Health Spending per Gross Domestic Product; PPHS/GDP – Prepaid Private Health Spending per Gross Domestic Product; OOPHS/GDP – Out-of-pocket Health Spending per Gross Domestic Product; DAH/GDP – Development Assistance for Health per Gross Domestic Product; GHS/THS

– Government Health Spending per Total Health Spending; PPHS/THS – Prepaid Private Health Spending per Total Health Spending; OOPHS/THS – Out-of-pocket Health Spending per Total Health Spending; DAH/THS – Development Assistance for Health per Total Health Spending.

Table S5. Direct and Indirect effects decomposition during short-term of HLE associated and health financing by using different spatial weight matrix in West Africa, 1995-2019.

Health financing component		Variables	Direct	Indirect	Total
Health financing level	Overall level	THS	-3.76(-6.99,-0.52)‡	-6.9(-14.27,0.47)	-10.66(-19.89,-1.42)‡
		GHS	0.47(-0.69,1.64)	3.44(0.54,6.35)‡	3.92(0.44,7.39)‡
		PPHS	-0.17(-0.62,0.29)	0.18(-0.73,1.1)	0.01(-1,1.03)
		OOPHS	3.86(0.87,6.85)‡	6.08(-0.44,12.6)	9.94(2.07,17.81)‡
		DAH	0.34(-0.1,0.77)	-1(-1.82,-0.18)‡	-0.66(-1.7,0.37)
	Per capita level	THS/PC	-0.03(-0.11,0.05)	-0.01(-0.17,0.15)	-0.04(-0.23,0.16)
		GHS/PC	0.02(-0.05,0.1)	-0.02(-0.19,0.16)	0.01(-0.2,0.22)
		PPHS/PC	0.1(-0.02,0.22)	0.3(0.05,0.55)‡	0.4(0.11,0.69)‡
		OOPHS/PC	0.02(-0.06,0.09)	-0.02(-0.19,0.15)	-0.01(-0.21,0.19)
		DAH/PC	0.02(-0.06,0.09)	0.01(-0.14,0.17)	0.03(-0.16,0.22)
	Per GDP level	THS/GDP	5.1(-4.34,14.54)	0.65(-17.89,19.18)	5.75(-15.37,26.87)
		GHS/GDP	0.54(-13.99,15.06)	12.95(-15.13,41.03)	13.48(-20.32,47.29)
		PPHS/GDP	-21.09(-54.95,12.76)	-48.44(-125.43,28.56)	-69.53(-167.67,28.61)
		OOPHS/GDP	-1.68(-14.28,10.93)	-11.08(-32.49,10.33)	-12.76(-38.31,12.8)
		DAH/GDP	11.53(-0.31,23.36)	9.94(-15.56,35.44)	21.47(-8.52,51.45)
Health financing structure	Government	GHS/THS	-0.78(-8.4,6.83)	-2.93(-17.24,11.39)	-3.71(-21.45,14.03)
	Nongovernment	PPHS/THS	-3.93(-13.51,5.64)	-17.18(-34.41,0.05)	-21.12(-43.01,0.78)
		OOPHS/THS	-3.03(-10.44,4.37)	0.37(-13.57,14.31)	-2.66(-19.92,14.59)
	External assistance	DAH/THS	-1.36(-8.66,5.95)	2.43(-10.96,15.81)	1.07(-15.53,17.67)

‡$P<0.05$; THS – Total Health Spending; GHS – Government Health Spending; PPHS – Prepaid Private Health Spending; OOPHS – Out-of-pocket Health Spending; DAH – Development Assistance for Health; THS/PC – Total Health Spending per person; GHS/PC – Government Health

Spending per person; PPHS/PC – Prepaid Private Health Spending per person; OOPHS/PC – Out-of-pocket Health Spending per person; DAH/PC – Development Assistance for Health per person; THS/GDP – Total Health Spending per Gross Domestic Product; GHS/GDP – Government Health Spending per Gross Domestic Product; PPHS/GDP – Prepaid Private Health Spending per Gross Domestic Product; OOPHS/GDP – Out-of-pocket Health Spending per Gross Domestic Product; DAH/GDP – Development Assistance for Health per Gross Domestic Product; GHS/THS – Government Health Spending per Total Health Spending; PPHS/THS – Prepaid Private Health Spending per Total Health Spending; OOPHS/THS – Out-of-pocket Health Spending per Total Health Spending; DAH/THS – Development Assistance for Health per Total Health Spending.
